# Attenuated Resting-State Functional Anticorrelation between Attention and Executive Control Networks in Schizotypal Personality Disorder

**DOI:** 10.3390/jcm10020312

**Published:** 2021-01-15

**Authors:** Ji-Won Hur, Taekwan Kim, Kang Ik K. Cho, Jun Soo Kwon

**Affiliations:** 1 Department of Psychology, Korea University, Seoul 02841, Korea; j_hur@korea.ac.kr; 2 Department of Brain and Cognitive Sciences, Seoul National University College of Natural Sciences, Seoul 08826, Korea; takwan99@snu.ac.kr; 3 Department of Psychiatry, Brigham and Women’s Hospital, Harvard Medical School, Boston, MA 02215, USA; kevincho@bwh.harvard.edu; 4 Department of Psychiatry, Seoul National University College of Medicine, Seoul 03080, Korea; 5 Institute of Human Behavioral Medicine, SNU-MRC, Seoul 03080, Korea

**Keywords:** schizotypal personality disorder, resting-state networks, resting-state functional connectivity

## Abstract

Exploring the disruptions to intrinsic resting-state networks (RSNs) in schizophrenia-spectrum disorders yields a better understanding of the disease-specific pathophysiology. However, our knowledge of the neurobiological underpinnings of schizotypal personality disorders mostly relies on research on schizotypy or schizophrenia. This study aimed to investigate the RSN abnormalities of schizotypal personality disorder (SPD) and their clinical implications. Using resting-state data, the intra- and inter-network of the higher-order functional networks (default mode network, DMN; frontoparietal network, FPN; dorsal attention network, DAN; salience network, SN) were explored in 22 medication-free, community-dwelling, non-help seeking individuals diagnosed with SPD and 30 control individuals. Consequently, while there were no group differences in intra-network functional connectivity across DMN, FPN, DAN, and SN, the SPD participants exhibited attenuated anticorrelation between the right frontal eye field region of the DAN and the right posterior parietal cortex region of the FPN. The decreases in anticorrelation were correlated with increased cognitive–perceptual deficits and disorganization factors of the schizotypal personality questionnaire, as well as reduced independence–performance of the social functioning scale for all participants together. This study, which links SPD pathology and social functioning deficits, is the first evidence of impaired large-scale intrinsic brain networks in SPD.

## 1. Introduction

Over the last two decades, there has been much interest in aberrant neural circuits associated with clinical symptoms and functional impairments of schizophrenia-spectrum disorders [1,2]. Of the various forms of schizophrenia-related disorders, schizotypal personality disorder (SPD) is assumed to lie at one end of the schizophrenia spectrum [3].

SPD is characterized by a persistent pattern of interpersonal deficits, paranoid ideation, magical thinking, unusual perceptual experiences, and eccentric behavior [4,5]. Given that it shares phenomenological and physiological characteristics with schizophrenia [6,7], it took a considerable amount of time for the SPD to settle in a suitable nosological position. Indeed, several neurophysiological studies involving individuals with schizotypal personality traits found volumetric reductions of their cortical areas [8] and exacerbated amphetamine-induced dopamine function [9]; these were analogous to the findings from schizophrenia research [7]. However, SPD can be distinguished from other diagnoses in the schizophrenia spectrum range since it has no identifiable psychotic symptoms, and it is now well-known that most individuals with SPD do not develop full-blown psychosis [4]. Naturally, the hypothesis that SPD is a precursor or risk factor for schizophrenia is now less persuasive [10]. As such, the schizotypal personality-specific clinical symptoms and their underlying neural correlates are now being extensively investigated.

Recent literature, driven by advances in neuroimaging research, argues that resting-state fMRI signals provide an optimal predictor of cognitive functions as well as the level of adaptation to the real world of individuals with schizophrenia spectrum disorders [11,12,13]. Network analysis is a robust way to investigate the pathophysiology of wide-range resting-state brain connectivity. Both the individual large-scale resting-state network (RSN), generated from the blood-oxygen-level-dependent (BOLD) signal at rest, and the fine balance between these RSNs are crucial for integrating internal and external environmental stimuli into the higher-order cognitive units [14,15]. For schizophrenia spectrum disorders, reduced coordination through RSNs relates to the failure to accomplish goal-oriented tasks [16,17].

In this study, we investigated potential alterations in the spontaneous neural activity at rest in individuals with SPD, focusing on the coupling between networks of interest, the default mode network (DMN), the frontoparietal network (FPN), the dorsal attention network (DAN), and the salience network (SN). These were termed “higher-order functional networks” in previous schizophrenia research because of their distinctive role in cognitive functions [18]. For instance, the DMN is activated during internally elicited thought, such as self-referential processes. Conversely, the FPN, SN, and DAN are responsible for the allocation of mental resources for externally directed cognition [19]. The SN is also believed to respond to a wide range of salient information, including cognitive or emotional information [16], while the DAN involves top-down attentional-control processes [20]. The FPN also associates cognitive control to the external world [16]. These networks are closely related to their activation patterns; for example, when the FPN is more negatively coupled with the DAN, the DAN becomes more negatively coupled with the DMN in healthy populations [21].

To the researchers’ knowledge, no study has examined the dynamics of inter-network coupling in SPD and their link with the schizotypal personality traits, while emerging findings suggest that the disruption of RSNs reflects the pathology of schizophrenia spectrum disorders [22,23]. Previous observations of RSNs in SPD have only been reported from healthy subjects with high “schizotypy” scores [24,25] or small sample sizes of subjects [26,27]. However, unlike the SPD, schizotypy is a nonpathological construct that could be seen in healthy populations. Furthermore, a review of the literature by Schultze-Lutter and colleagues [28] concluded that benign schizotypy appears to share the same dimensions as schizophrenia rather than SPD, and, therefore, schizotypy and SPD are on qualitatively different dimensions. Hence, further refinements in methodology for SPD research reflecting its genuine psychopathology are inevitable. In this study, the inclusion criteria for research participants were restricted to community-dwelling, non-help seeking subjects who were diagnosed as having SPD by professional clinicians. In following these criteria, we expected to uncover SPD-specific pathophysiology. We also hypothesized that imbalanced intrinsic networks of SPD correlate with their social functioning deficits, as well as schizotypal personality symptoms.

## 2. Methods and Materials

### 2.1. Participants

All participants were recruited via flyers, online postings, and word of mouth. Potential participants for the SPD group (*n* = 250) were screened via structured phone interviews. Of the 250 volunteers, 65 eligible individuals were interviewed face-to-face using the Structured Clinical Interview for DSM-IV-Non-Patient Edition (SCID-NP) and the Structured Clinical Interview for DSM-IV Personality Disorders (SCID-II) by psychiatrists and a licensed clinical psychologist. Finally, 22 community-dwelling, non-help seeking individuals who were diagnosed with SPD and 30 controls matched for age, sex, handedness, IQ, education, and socioeconomic status (SES) participated in this study. All participants had corrected or normal vision, and had no history of psychosis, substance use disorders or neurological disorders, current major depressive disorder, antipsychotics use, or family history of major psychiatric illness. The recruitment process has been described in detail previously [29]. Due to the participants’ head motion and sleepiness levels during the resting scan, the number of subjects was not the same. The short-form of the Korean version of the Wechsler Adult Intelligence Test was also conducted to estimate participants’ general intelligence [30], and there were no group differences in IQ estimates (Table 1).

### 2.2. Measures

#### 2.2.1. Clinical Symptoms

The Korean version of the Schizotypal Personality Questionnaire (SPQ) was used to assess the schizotypal personality traits of all participants [31,32]. The SPQ is a 4-point Likert scale containing 74 items that reflect the nine DSM-IV-TR criteria for SPD: ideas of reference (e.g., Have you ever noticed a common event or object that seemed to be a special sign for you?), social anxiety (e.g., I feel very uncomfortable in social situations involving unfamiliar people.), odd beliefs or magical thinking (e.g., Are you sometimes sure that other people can tell what you are thinking?), unusual perceptual experiences (e.g., I often hear a voice speaking my thoughts aloud.), odd or eccentric behavior (e.g., People sometimes comment on my unusual mannerisms and habits.), no close friends (e.g., I prefer to keep myself to myself.), odd speech (e.g., People sometimes find it hard to understand what I am saying.), constricted affect (e.g., People sometimes find me aloof and distant.), and suspiciousness/paranoid ideation (e.g., Have you found that it is best not to let other people know too much about you?).

The SPQ also includes the three factors of the schizotypal personality: cognitive–perceptual deficits (ideas of reference, odd beliefs or magical thinking, and unusual perceptual experiences), interpersonal deficits (social anxiety, no close friends, constricted affect, and suspiciousness/paranoid ideation), and disorganization (odd or eccentric behavior and odd speech). The SPQ has adequate reliability and validity as an index of this construct [31,33].

The overall psychological disturbance was measured using the Global Assessment of Functioning Scale (GAF) [34].

#### 2.2.2. Social Functions

Reading the Mind in the Eyes Test (RMET) [35,36] assessed participants’ emotion perception abilities. This 4-option multiple-choice test consists of 36 photographs of the eye region embedding different emotional valences (8 positive, 12 negative, and 16 neutral expressions). In this study, all subjects were requested to infer a mental state from each RMET trial, presented via Psychopy, by pressing a button on the keyboard.

The social skills and performance of participants were assessed by the Social Functioning Scale (SFS), which is a self-administered questionnaire composed of 79 items [37]. The SFS is divided into seven dimensions: (1) social engagement/withdrawal (time spent alone and initiation of conversations and social avoidance), (2) interpersonal behavior (number of friends, presence of a romantic partner, and ability to start conversations), (3) prosocial activities (passive or active engagement in a range of social events, such as cinema or sport), (4) recreational activities (involvement in a variety of solitary activities, such as hobbies, interests, or pastimes), (5) independence–competence (ability to perform skills necessary for independent living), (6) independence–performance (performance in skills needed for independent living), and (7) employment/occupation (engagement in productive employment or a structured daily program). The standardized scores for each subscale were used, and higher scores of the SFS indicate more competent behaviors or higher performance levels. The SFS has been demonstrated to have good internal consistency [37].

### 2.3. Image Acquisition and Preprocessing

We used a 3T magnetic resonance imaging (MRI) scanner (Siemens Magnetom Trio, Erlangen, Germany) with a 16-channel head coil at Seoul National University to acquire functional and structural brain images. We scanned resting-state functional imaging data using a gradient echo-planar imaging pulse sequence for 6 min and 58 s with the following parameters: repetition/echo time = 3500/30 ms, flip angle = 90°, 1.9 × 1.9 × 3.5 mm voxel dimension, and 35 axial slices acquired in interleaved sequence. Participants were instructed to relax with their eyes open, but not to fall asleep during the resting-state session. We also acquired T1-weighted anatomical images using a magnetization-prepared rapid-gradient echo sequence with the following parameters: repetition/echo time = 1670/1.89 ms, flip angle = 9°, 1 mm isotropic voxels, and 208 sagittal slices.

We preprocessed the brain imaging data using the Statistical Parametric Mapping toolbox version 12 (SPM12, UCL Queen Square Institute of Neurology, London, UK; http://www.fil.ion.ucl.ac.uk/spm/). The anatomical brain images were segmented into different tissue types. For the resting-state fMRI data, we discarded the first four volumes and corrected slice timing. We realigned the functional images to the first image and estimated head motion via rigid-body translation. Eight SPD subjects and 9 controls with excessive head motion (larger than 1 mm translation or 1° rotation, in any direction) were excluded from the subsequent analysis. The functional images were coregistered to the anatomical images. Subsequently, all brain images were normalized to the Montreal Neurological Institute (MNI) space with an isotropic voxel size of 2 mm [38]. We spatially smoothed the functional images with a 6 mm full width at half-maximum Gaussian kernel and cleaned noise signals with the aCompCor method, linear detrending, and temporal band-pass filtering [39].

### 2.4. Intra- and Inter-Network Functional Connectivity

To investigate intra- and inter-network functional connectivity, we used canonical RSNs that were estimated from independent component analysis (ICA) of the Human Connectome Project dataset, which had 497 participants and strong empirical support [40,41,42]. In this study, we selected the DMN, SN, DAN, and FPN, according to the previous findings from schizophrenia spectrum research [18]. The regions of interest (ROIs) for DMN, FPN, DAN, and SN comprised the subregions of each network. In this study, we constructed a 19 × 19 connectivity matrix and measured the connectivity strength using Pearson’s correlation; the correlation coefficients were converted to a normal distribution using Fisher’s *z* transformation. The coordinates of each subregion are described in Supplementary Table S1.

### 2.5. Statistical Analyses

In this study, we compared intra- and inter-network connectivity differences between groups using a general linear model (GLM). We named the correlation between two ROIs in the same network as “intra-network connectivity” and the correlation between ROIs from two different networks as “inter-network connectivity.” First, the mean signal time series were extracted from the 19 ROI of 4 RSNs of each subject using the CONN toolbox. A two-sample *t*-test was then used to compare the mean ROI-to-ROI connectivity matrices between groups. False-positive control was implemented by applying the connection-level false-discovery rate (FDR)-corrected *p* < 0.05. A connectogram was generated using Circos software (https://genome.cshlp.org/citmgr?gca=genome;gr.092759.109v1; http://www.circos.ca) to visualize overall connectivity across all RSN regions during rest. Additionally, we performed a seed-based resting-state fMRI analysis using the ROIs defined as regions associated with altered inter-network connectivity aimed to further investigate whether the positive or negative direction of the correlations found in the inter-network connectivity indicated an actual positive or anticorrelated connectivity at the seed to voxel level. Demographic and clinical variables were also analyzed using an independent two-sample *t*-test or chi-squared test.

## 3. Results

### 3.1. Participant Characteristics

The demographic and clinical characteristics are summarized in Table 1. Clinical and neuropsychological measures showed that individuals with SPD displayed higher scores on all clinical measures, including SPQ total score (*t* = 9.68, *p* < 0.001) and GAF score (*t* = −8.36, *p* < 0.001) compared to controls, but had comparable RMET performance (*t* = −1.34, *p* = 0.19).

### 3.2. Overall Characteristics of Intra- and Inter-Network Connectivity

**Figure 1:**
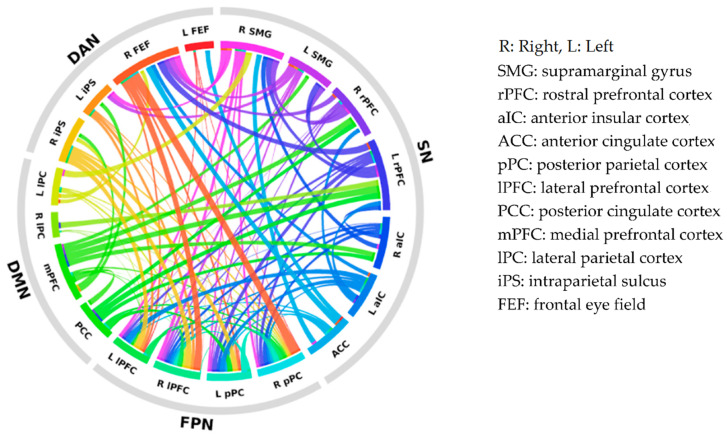
Connectogram of intra- and inter-network connectivity differences between subregions of the default mode network (DMN), the salience network (SN), the dorsal attention network (DAN), and the frontoparietal network (FPN). Each colored segment represents each subregion, and these are grouped according to resting-state networks. The lines connecting two segments represent the degree of connectivity strength differences between SPD and control participants. The thicker the line, the larger the strength difference. SPD: schizotypal personality disorder.

To graphically illustrate the group differences in intra- and inter-network connectivity, a connectogram of the RSN regions was created (see Figure 1). Each connection denotes the correlation between two brain regions and has a threshold at the upper 25% of the strongest connections for visualization purposes. The thicker the line, the larger the strength difference.

#### 3.2.1. Intra-Network Connectivity

**Figure 2:**
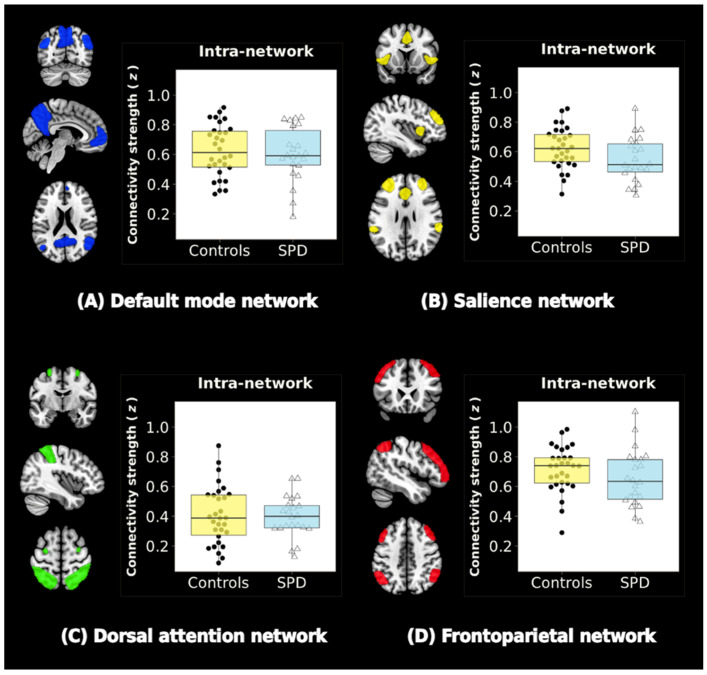
Resting-state networks (RSNs) and intra-network connectivity. Four RSNs are shown: (**A**) the default mode network (DMN), (**B**) the salience network (SN), (**C**) the dorsal attention network (DAN), and (**D**) the frontoparietal network (FPN). Color-bar graphs summarize the results of comparisons between SPD and control groups. Z-values indicate the functional connectivity strength within the network. There were no significant group differences in intra-network functional connectivity (FDR-corrected *p*-values > 0.05).

No significant group differences were observed within the DMN, SN, DAN, or FPN (FDR-corrected *p*-values > 0.05) (Figure 2).

#### 3.2.2. Inter-Network Connectivity

**Figure 3:**
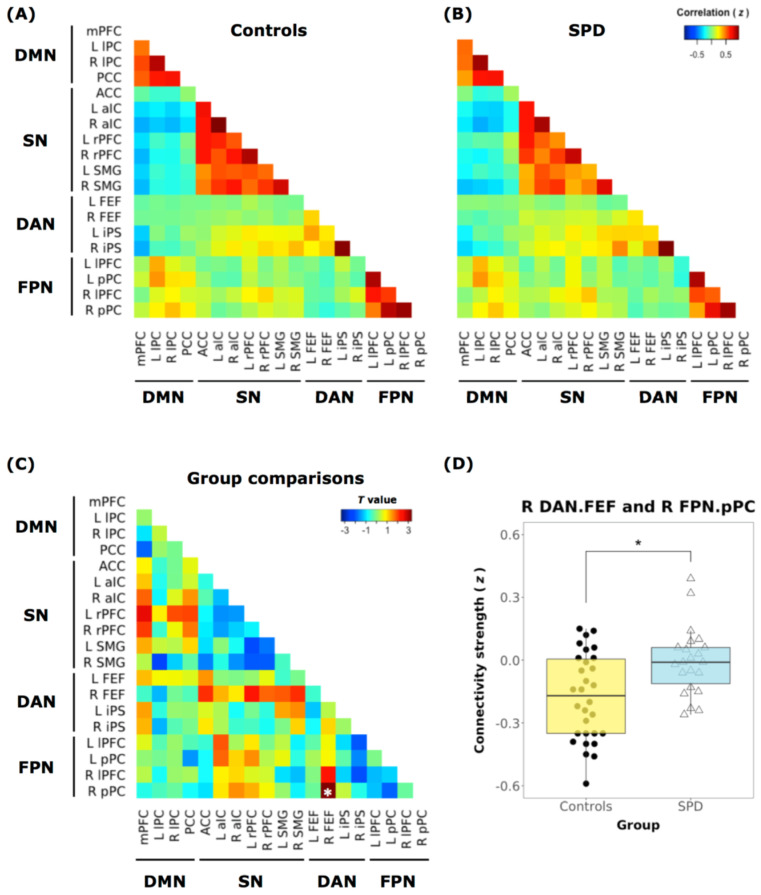
Inter-network connectivity matrices. Inter-network connectivity matrix for the (**A**) controls and (**B**) individuals with SPD. Network nodes in the functional matrix are arranged according to the 19 regions of interest (ROIs) from the 4 RSNs (DMN, SN, DAN, and FPN). (**C**) Mean connectivity difference between SPD participants and controls (*t*-value). The correlation coefficient (Fisher’s z) demonstrates attenuated connectivity strength (anticorrelation) between the right frontal eye field region (R FEF) of DAN and the right posterior parietal cortex region (RpPC) of FPN in participants diagnosed with SPD compared to the controls. Asterisks (∗) labeled on the matrix indicate FDR-corrected *p* < 0.05. (**D**) There is reduced anticorrelation between the DAN-FPN in the SPD group (right, blue bar) compared to the controls (left, yellow bar). A subnetwork pair between the RFEF of the DAN and the RpPC of FPN shows significant group differences (SPD vs. Controls). mPFC: medial prefrontal cortex, lPC: lateral parietal cortex, PCC: posterior cingulate cortex, ACC: anterior cingulate cortex, aIC: anterior insular cortex, rPFC: rostral prefrontal cortex, SMG: supramarginal gyrus, FEF: frontal eye field, iPS: intraparietal sulcus, lPFC: lateral prefrontal cortex, pPC: posterior parietal cortex.

In our DMN/SN/DAN/FPN large-scale network analyses, individuals with SPD showed weaker anticorrelated functional connectivity between the right frontal eye field region of the DAN and right posterior parietal cortex region of the FPN compared to controls (*t* = 3.19, FDR-corrected *p* = 0.04) (Figure 3).

### 3.3. Correlation Analysis

There was no correlation between a significant subnetwork pair and SPQ subscales within either group separately. However, across all participants, the weaker the anticorrelation between DAN and FPN, the more severe the ideas of reference (*r* = 0.37; *p* = 0.007), odd beliefs or magical thinking (*r* = 0.37; *p* = 0.007), unusual perceptual experiences (*r* = 0.37; *p* = 0.008), odd or eccentric behavior (*r* = 0.48; *p* < 0.001), and odd speech (*r* = 0.40; *p* = 0.004) subscales.

In terms of the SPQ factors, the reduced strength of the anticorrelation between DAN and FPN also correlated to the cognitive–perceptual deficits (*r* = 0.38; *p* = 0.006) and disorganization (*r* = 0.46; *p* = 0.001) factors of the SPQ in all participants.

**Figure 4:**
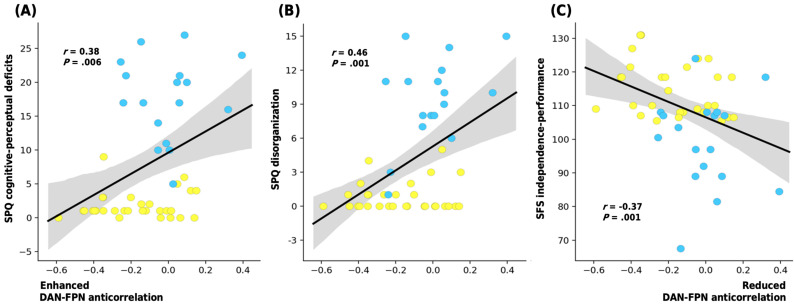
Plots displaying the correlation between the connectivity strength of R FEF of DAN and RpPC of FPN (the Z-value of peak DAN and FPN anticorrelation) with the participants’ (**A**) SPQ cognitive-perceptual deficits, (**B**) SPQ disorganization, and (**C**) SFS independence-performance scores. Shaded areas illustrate a 95% confidence interval; Pearson’s correlation coefficient (*r*) is also provided (SPQ: Schizotypal Personality Questionnaire; SFS: Social Functioning Scale, SPD: schizotypal personality disorder).

The attenuated anticorrelation also showed a negative correlation with the independence–performance subscale of the SFS (*r* = −0.37; *p* = 0.007) for all participants (Figure 4). There were no other significant correlations of the RSNs with the clinical traits or GAF scores.

## 4. Discussion

The prevalence of SPD, which threatens social adjustment and hampers the overall well-being of an individual, is 4.6% among the general population; therefore, it is a significant mental health issue [5]. However, compared to other schizophrenia spectrum disorders, little is known of the intrinsic system of brain networks among individuals with SPD and its clinical implications. In this study, we found that individuals diagnosed with SPD demonstrated decreased anticorrelation between the DAN, which serve voluntary “top-down” attention to goals, and FPN, known to be involved in adaptive executive control [43,44]; the control participants revealed an anticorrelation between the DAN and FPN. We also found that the weakened strength of the anticorrelation was linked to the *cognitive*–*perceptual deficits* and *disorganization* factors of SPQ, as well as the lower *independence*–*performance* subscale of SFS for all participants. To our knowledge, this is the first study to examine the communication of subcomponents of a large-scale brain network in individuals diagnosed with SPD and its implication on the core schizotypal personality abnormalities.

As the name reveals, the pivotal function of the DAN, modulated by frontal-to-parietal top-down streams [45], is voluntary attention control [46,47]. Superficially, the DAN appears similar to the dynamics of the FPN, where neural signals increase during cognitive tasks that require attention to external demands [48]. However, the top-down direction of the DAN, which prepares the organism to process perceptually or semantically salient stimuli, is less prominent during less attentive resting states [46]. As such, the DAN and FPN show a negative correlation (anticorrelation) in healthy adults [49] depending on how FPN subsystems engage [50].

We similarly found that the subcomponents of the DAN and FPN were negatively correlated among controls. This is also in line with previous work by Chai, Ofen, Gabrieli, and Whitfield-Gabrieli [51], which showed increased anticorrelations between the DAN and lateral parietal regions in healthy youth. Meanwhile, the anticorrelation between the FEF of DAN and pPC of FPN was significantly attenuated in individuals with SPD. The FEFs are the origin of the causal streams along the DAN, while the pPC is one of the key hubs of the FPN [46,47]. Consequently, the altered communication between these components may have cascading, detrimental effects on the optimal dynamics of RSNs involved with various cognitive processes ranging from perceptual–attentional to higher-order mental processes. In particular, impaired anticorrelations between the DAN and other networks reflect disrupted functional integration, which may lead to neurodevelopmental problems [52].

These findings are quite different from the previous literature, in which the altered functional connectivity between DMN and FPN [53] and DMN and SN [54,55] were reported in individuals with schizophrenia. The discrepancy between the previous results and our findings may reflect the fundamental difference between schizophrenia and schizotypal personality disorder. In particular, the abnormal functional network dynamics in full-blown psychosis have been identified as the neural correlates of the psychopathology of psychosis, such as hallucinations [53,55]. However, this should be interpreted with caution since no direct comparison of brain dynamics in schizophrenia versus schizotypal personality disorder has been made in the current study.

Of particular interest is how alterations in RSNs are linked to the clinical characteristics of SPD. Across all participants, we found that the attenuated anticorrelation between the DAN and FPN in SPD demonstrated a moderate correlation with increased cognitive–perceptual deficits (e.g., ideas of reference, magical thinking, and unusual perceptual experiences) and disorganization factor (e.g., eccentric behavior and odd speech) on the SPQ. Intriguingly, these two factors of the SPQ, except the interpersonal deficits factor, are closely associated with self-reported everyday executive problems in schizotypal participants [56]. The correlation results appear to be aligned with previous studies that viewed cognitive–perceptual deficits and disorganization as core features of SPD in that interpersonal deficits are more common in the general population or cluster C personality disorders (also known as anxious–fearful) rather than SPD [57,58]. On the other hand, there was no significant correlation in the separate analyses within each group. This was probably because of the reduced sample size compared to the correlation analysis for the whole group. However, our findings need to be replicated with a larger number of samples in future studies.

Our finding of abnormal network connectivity involving the failure of the higher-order cognitive processes also provides a neurophysiological account of the maladaptive daily behaviors of SPD. In the correlation analysis with the *independence–performance* subscale of the SFS, it was found that the weak anticorrelation of RSNs was related to the lack of skill performance required for individuals’ independent living. According to Dixson and his colleagues, the disruption of anticorrelation between the DAN and FPN implicates abnormalities in the integration of attentional resources [50], which leads to a failure in abstract thinking [21]. Taken together, the present results indicate that the altered RSNs of SPD may be a promising biomarker that reflects both the core symptoms of SPD and the everyday behavioral problems that occur in relation to attentional control of the limited cognitive resources to the environmental cues [59].

Several strengths and limitations should be acknowledged. The major strength of this study is that all SPD group data were acquired from participants diagnosed with SPD to uncover SPD-specific neuropathology. It was also guaranteed that the SPD participants retained appropriate cognitive resources (mean IQ 119.3 ± 7.4). Therefore, we can exclude the effect of impairments of general intelligence, a main confounding factor in psychosis studies [60], on the dependent variables. Another strength is that all non-help seeking, community-dwelling participants were not receiving any medication; our results may thus provide a better understanding of the pathogenesis of SPD by excluding possible confounders. Limitations include the analysis’s limitation to a priori ROIs, which are implicated in the RSNs. Further studies using a bottom-up, data-driven approach may expand our findings on biomarkers for SPD. Additionally, we did not confirm a significant correlation between brain activity and clinical measures within the SPD group. The current findings using all participants could be generalizable in that schizophrenia spectrum disorders are regarded as being on a continuum that begins with normality and proceeds towards mental illness; however, in future studies, it may be necessary to confirm the characteristics of individuals with SPD by using larger samples and various scales.

## 5. Conclusions

In conclusion, we found that an anticorrelation between the DAN and the FPN at rest observed for controls was attenuated in SPD participants. Notably, we provided substantial evidence for the significant association between altered intrinsic functional network dynamics and schizotypal personality symptoms and impaired social functioning. The present study is the first to examine abnormalities in the large-scale resting-state dynamics of medication-free, non-help seeking individuals who were diagnosed with SPD. This study further extends previous SPD psychopathology studies and provides a helpful reference for future applications of neuromodulation for the intervention of SPD.

## Figures and Tables

**Table 1:** Demographic, clinical, and psychological characteristics.

Variables	SPD	Controls	Statistics	
	(*N* = 22)	(*N* = 30)	*t* (*χ*^2^)	*p*-Value
Age, mean (SD), years	22.68 (3.71)	22.53 (2.60)	0.16	0.87
Sex, male/female	15/7	23/7	(0.46)	0.50
Handedness, left/right/ambidextrous	18/4/0	28/1/1	(3.83)	0.15
Estimated IQ, mean (SD)	119.32 (7.40)	120.10 (7.14)	−0.38	0.70
Education, mean (SD), years	14.86 (1.55)	14.90 (1.06)	−0.10	0.92
SES self, mean (SD)	3.05 (1.17)	2.67 (0.80)	(3.61)	0.46
SES parental, mean (SD)	2.86 (0.99)	2.77 (0.77)	(3.01)	0.56
SPQ total, mean (SD)	35.05 (13.61)	5.37 (5.44)	9.68	<0.001
Subscale 1: Ideas of reference	5.24 (2.51)	1.10 (1.06)	7.13	<0.001
Subscale 2: Social anxiety	3.95 (2.29)	1.50 (1.78)	4.31	<0.001
Subscale 3: Odd beliefs/magical thinking	3.86 (1.68)	0.10 (0.31)	10.12	<0.001
Subscale 4: Unusual perceptual experiences	4.00 (2.24)	0.10 (0.31)	7.94	<0.001
Subscale 5: Eccentric/odd behavior and appearance	4.05 (2.06)	0.20 (0.48)	8.40	<0.001
Subscale 6: No close friends	3.48 (2.48)	0.50 (0.90)	5.26	<0.001
Subscale 7: Odd speech	4.86 (2.37)	0.63 (1.03)	7.67	<0.001
Subscale 8: Constricted affect	3.24 (1.48)	0.77 (1.25)	6.44	<0.001
Subscale 9: Suspiciousness/paranoid ideation	3.62 (1.86)	0.53 (1.07)	6.86	<0.001
SPQ factor 1: Cognitive–perceptual deficits	16.71 (6.37)	1.83 (2.05)	10.33	<0.001
SPQ factor 2: Interpersonal deficits	14.29 (6.45)	3.30 (3.69)	7.04	<0.001
SPQ factor 3: Disorganization	8.90 (3.90)	0.83 (1.34)	9.12	<0.001
GAF, mean (SD)	66.00 (13.58)	90.70 (3.20)	−8.36	<0.001
RMET (correct), mean (SD)	25.55 (2.81)	26.50 (2.32)	−1.34	0.19
SFS total, mean (SD)	102.67 (10.78)	116.39 (4.84)	−6.19	<0.001
Withdrawal	98.98 (11.32)	112.62 (9.34)	−4.75	<0.001
Interpersonal	108.14 (16.57)	120.40 (12.27)	−3.07	0.003
Prosocial	103.57 (15.48)	120.58 (8.82)	−4.64	<0.001
Recreation	98.55 (12.97)	110.43 (14.33)	−3.08	0.003
Independence–competence	93.66 (24.21)	113.72 (9.36)	−4.15	<0.001
Independence–performance	100.30 (12.82)	115.20 (8.33)	−5.08	<0.001
Employment/occupation	115.52 (11.92)	121.78 (2.21)	−2.43	0.024

SPD: schizotypal personality disorder; SES: socioeconomic status; SPQ: Schizotypal Personality Questionnaire; GAF: Global Assessment of Functioning Scale; RMET: Reading the Mind in the Eyes Test; SFS: Social Functioning Scale; SD: standard deviation.

## Data Availability

The data that support the findings of this study are available on request from the corresponding author. The data are not publicly available due to privacy or ethical restrictions.

## Supplementary Materials

The following are available online at https://www.mdpi.com/2077-0383/10/2/312/s1, Table S1: Montreal Neurological Institute (MNI) coordinates of resting-state network subregions.
