# Supplementary material for: Attenuated Resting-State Functional Anticorrelation between Attention and Executive Control Networks in Schizotypal Personality Disorder
Source: J Clin Med. 2021 Jan 15;10(2):312. doi: 10.3390/jcm10020312 (PMC7829946; doi:10.3390/jcm10020312)
Supplement: Supplementary file 1 [file jcm-10-00312-s001.pdf]

Table S1. Montreal Neurological Institute (MNI) coordinates of resting-state network subregions.

Networks	Subregions	MNI Coordinates (mm)		
		x	y	z
DMN	mPFC	1	55	−3
	L lPC	−39	−77	33
	R lPC	47	−67	29
	PCC	1	−61	38
SN	ACC	0	22	35
	L aIC	−44	13	1
	R aIC	47	14	0
	L rPFC	−32	45	27
	R rPFC	32	46	27
	L SMG	−60	−39	31
	R SMG	62	−35	32
DAN	L FEF	−27	−9	64
	R FEF	30	−6	64
	L iPS	−39	−43	52
	R iPS	39	-42	54
FPN	L lPFC	−43	33	28
	L pPC	−46	−58	49
	R lPFC	41	38	30
	R pPC	52	−52	45

DMN, default mode network; SN, salience network; DAN, dorsal attention network; FPN, frontoparietal network; mPFC, medial prefrontal cortex; lPC, lateral parietal cortex; PCC, posterior cingulate cortex; ACC, anterior cingulate cortex; aIC, anterior insular cortex; rPFC, rostral prefrontal cortex; SMG, supramarginal gyrus; FEF, frontal eye field; iPS, intraparietal sulcus; lPFC, lateral prefrontal cortex; pPC, posterior parietal cortex.
